# The Effect of d^10^ Precious Elements on Structural, Magnetic and Elastic Properties of MnPt Alloy: A First-Principles Study

**DOI:** 10.3390/ma17030541

**Published:** 2024-01-23

**Authors:** Ramogohlo Diale, Phuti Ngoepe, Hasani Chauke, Joseph Moema, Maje Phasha

**Affiliations:** 1 Advanced Materials Division, Mintek, Private Bag X 3015, Randburg 2125, South Africa; josephm@mintek.co.za (J.M.); majep@mintek.co.za (M.P.); 2 Materials Modelling Centre, University of Limpopo, Private Bag X 1106, Sovenga 0727, South Africa; phuti.ngoepe@ul.ac.za (P.N.); hasani.chauke@ul.ac.za (H.C.)

**Keywords:** Mn_50_Pt_50−x_M_x_ (M = Pd; Au; Ag), magnetic properties, elastic properties, density functional theory

## Abstract

MnPt’s exceptional stability and extremely high Néel temperature have generated a lot of interest in data storage applications. Previously, it was reported experimentally that the MnPt alloy shows ferromagnetic (FM) behavior at room temperature. In this study, the effects of partial substitution of Pt with Pd, Au, and Ag on magnetic properties is investigated using density functional theory. The stability of Mn_50_Pt_50−x_M_x_ (M = Pd, Au, Ag, x = 6.25, 12.5, 18.75) alloys was assessed by determining their thermodynamic, magnetic, and mechanical properties. The calculated lattice constants of Mn_50_Pt_50_ agree well with available theoretical results. The Mn_50_Pt_50−x_M_x_ alloys’ formability was assessed by measuring the thermodynamic stability using the heat of formation. It was found that B2 Mn_50_Pt_50−x_Pd_x_ alloys (0 ≤ x ≤ 18.75) are thermodynamically stable due to the negative heat of formation close to that of a pristine MnPt alloy. Based on the elasticity results, the B2 Mn_50_Pt_50−x_Pd_x_ is most likely to undergo martensitic transformation for the entire considered composition range. From the calculated values of the Poisson′s ratio, it is shown that an increase in Pd, Ag, and Au effectively improves the ductility of the B2 Mn_50_Pt_50−x_M_x_ compounds. It was revealed that ferromagnetism is maintained with Pd addition but significantly reduced in the case of Au and Ag. Thus, this work showed that density functional theory can be exploited to propose new possible compositions for future magnets in spintronic applications.

## 1. Introduction

Manganese (Mn) has received considerable attention in the past few years due to its low cost as a result of its abundance in the earth’s crust as well as its fascinating magnetic properties, which emerge when the separation distance between its atoms is increased by the presence of elements with either larger atomic radius or higher melting point to form an ordered intermetallic phase [1]. As a result of their half-filled 3d orbitals, most Mn-based intermetallic compounds formed at near-equiatomic composition with elements belonging to groups 9 and 10 of the periodic table reveal a typical antiferromagnetic (AFM) characteristic [2,3]. It is this characteristic and other properties, such as high stability of antiferromagnetism as well as very high Néel temperature (*T_N_*), that have aroused significant interest in AFM materials such as NiMn, IrMn, PdMn, RhMn and MnPt for their potential practical applications in the microelectronics industry. In contrast to conventional spintronic devices which use FM as the active element, the anti-ferromagnet spintronics use the AFM as the active element to store, read or write data much faster. One of the major attractive points of AFM materials is their insensitiveness to parasitic electromagnetic and magnetic interference [4]. However, the magnetization of AFMs is very difficult to manipulate [5]. As deployed successfully in giant magnetoresistance (GMR) sensors, the best of both is achieved by coupling the AFM to an FM. This vital exchange biasing between AFM and ferromagnetic (FM) layers in thin films results in the pinning effect by the AFM on an FM in magnetoresistance sensor and spin-valve heads [6]. The chief goal in this research and development effort is to achieve energy-efficient data storage by, in principle, finding a substitute for hard disk drives (HDD) and highly volatile dynamic random-access memory (DRAM) with a better multi-tasking performance at lower power consumption [7].

Among the above AFM material contenders, MnPt is the only one that exhibits the ferromagnetic exchange interactions characterized by out-of-plane equilibrium spin texture [2,4]. Due to its high ferromagnetic stability, it possesses excellent properties such as large exchange coupling fields and high blocking temperatures [8,9,10]. The equiatomic MnPt consists of the ordered paramagnetic cubic structure of type B2 (Pm-3m), which is transformed to a tetragonal structure of type L1_0_ (CuAu-I) at 970 K. AFM coupling between adjacent Mn atoms in the (100) plane at a distance and ferromagnetic coupling between adjacent Mn atoms in the [001] direction account for the AFM ordering in MnPt experiments [11]. On the other hand, although it is difficult to process, the formation of FM phases is scarcely reported to occur in the MnPt samples, resulting in disordered and partially ordered Mn atoms [8]. A lot of interest has been taken in MnPt alloys lately since the type of magnetic interaction can be studied based on degree of order, atom separation, and even environmental factors [12]. Moreover, since it is known that the magnetic moment of Mn is higher than that of ferromagnetic metals such as iron (Fe), cobalt (Co) and nickel (Ni), research on how this FM aspect can be exploited for technological benefits is necessary.

In addition to magnetic recording and spintronic applications, MnPt permalloys can also be used as FM active elements instead of AFM active elements in magnetic random-access memory (MRAM). Magnetic susceptibility and electrical resistivity of MnPt alloys have been reported at 1220 K [13,14]. This viewpoint also brings about the possibility of having an exchange bias between FM and AFM comprised of only MnPt alloys. However, the processing of the binary MnPt alloys remains a huge obstacle to this possibility. The structural, electronic, and magnetic properties of the ordered binary L1_0_ FePt, MnPt, and CrPt_3_ alloys were determined using the density functional theory (DFT) approach in a previous study [15]. It was found that L1_0_ MnPt exhibits magnetocrystalline anisotropy energy (MAE) values of 0.46 meV/f.u (1.3 × 10^7^ erg/cm^3^). It has been found that the smaller MAE value of L1_0_ MnPt can be attributed to weak hybridization between Mn-3d and Pt-5d states. Due to its capability to provide insightful information on materials behavior, this theoretical platform can also be used to search for suitable alloying elements that can stabilize the FM MnPt, thus easing the processing challenges.

In this regard, the current authors have previously reported the effect of antiferromagnetic (Cr) and ferromagnetic (Fe) elements on MnPt alloy using the DFT-based first-principles calculations [16]. It was discovered that the inclusion of Fe causes the magnetic moment to fall below that of Pt_50_Mn_50_, while the magnetism is enhanced when c/a is 1.10 for Pt_50_Mn_43.75_Cr_6.25_. The L1_0_ phase was preferred over the B2 phase by both Fe and Cr on the Mn-site, as demonstrated by the thermodynamic and mechanical stability. Furthermore, in another study, we investigated the effect of ferromagnetic element (Co) on the magnetic properties of Mn_50_Pt_50_ alloy using the same theoretical approach [17]. The findings indicated that because of the negative heats of formation, the addition of Co favored L1_0_ over the B2 phase. The study revealed that the addition of Co on both B2 and L1_0_ Mn_50_Pt_50_ results in improved magnetic strength and mechanical stability on the Pt-site. Based on the findings above, it is clear that magnetic strength and mechanical stability can be improved on the Pt-site as compared to the Mn-site. While most first-principles studies have focused on AFM MnPt, the current focus is on an attempt to stabilize FM MnPt-X in the B2 phase.

In the current work, the ab initio DFT approach was used to investigate the effect of the d^10^ elements (Pd, Au, Ag) on the structural, thermodynamic, magnetic and elastic properties of Mn_50_Pt_50−x_M_x_ ternary compositions. The choice of these alloying elements was based on our interest in studying the effect of introducing elements with larger atomic radii on the Pt-site. The rest of this paper is organized as follows. The theoretical method of calculation is provided in Section 2, and the results such as the heat of formation, magnetic moments, elastic constants and moduli are discussed in Section 3. Lastly, a conclusion is provided in Section 4.

## 2. Computational Method

In this paper, calculations on structural, magnetic, and elastic properties were performed using density functional theory (DFT) employing the Vienna ab initio simulation package (VASP) [18,19]. The Projector Augmented Wave (PAW) pseudopotentials were used to describe electron-ion interactions. The spin-polarized generalized gradient approximation (GGA) [20], parameterized by Perdew–Burke–Ernzerhof (PBE) [21], was used to account for the exchange-correlation functional. A spin polarization was included in our calculations. Regarding the number of k-points and the plane-waves basis set size, the convergence of the total energies was examined. The real ionic and valence electron interaction was described by ultra-soft pseudopotential based on the first-principles calculations. The total energy of the structures was converged using a 500 eV plane wave cutoff energy. We carried out sets of calculations of B2 Mn_50_Pt_50−x_M_x_ alloys (0 ≤ x ≤ 18.75) with a mesh grid of 14 × 14 × 14 to converge the total energy of the structures, according to Monkhorst and Pack [22]. For the force theorem calculation alone, the spin-orbit coupling was taken into consideration. A 2 × 2 × 2 supercell was used to perform the calculations. Using a substitutional search tool integrated into MedeA, Pt was replaced with Pd, Au, and Ag to produce the most stable compositions, which included 6.25, 12.50, and 18.75 at. % at the appropriate symmetry. The setting of “accurate” was applied throughout the calculation procedure to reduce errors and avoid wrap-arounds. Then, every structure was completely relaxed. Following the relaxed atomic positions, the total energy was minimized (i.e., as small as 10^−6^ eV) and the Hellmann–Feynman forces. In the unit cell of every electronic structure relaxation that was carried out, these forces were as small as 0.0003 eV/Å at convergence. A strain of 0.005 was used to calculate elastic constants for all structures. Using the Voigt approximation, the bulk modulus, shear modulus, elastic modulus, and Poisson ratio were consequently determined [23]. The paramagnetic and ferromagnetic B2 MnPt alloy phonon dispersion curves were assessed using the PHONON code [24] as implemented in Materials Design within the MedeA software platform, version 3.6. Each and every calculation was done at 0 K.

## 3. Results and Discussion

### 3.1. Structural and Magnetic Properties

In the MnPt alloy, the cubic B2 phase and the tetragonal L1_0_ crystal structure exist at high and low temperatures, respectively. The high temperature phase (B2) belongs to space group Pm-3m, No: 221 with the following Wyckoff atomic positions: Mn located at 1a (0, 0, 0) and Pt at 1b (1/2, 1/2, 1/2). The low temperature phase (L1_0_) belongs to space group P4/mmm, No: 123 with Wyckoff atomic position of Mn at 1a (0, 0, 0) and Pt at 1d (0, 1/2, 1/2).

**Figure 1:**
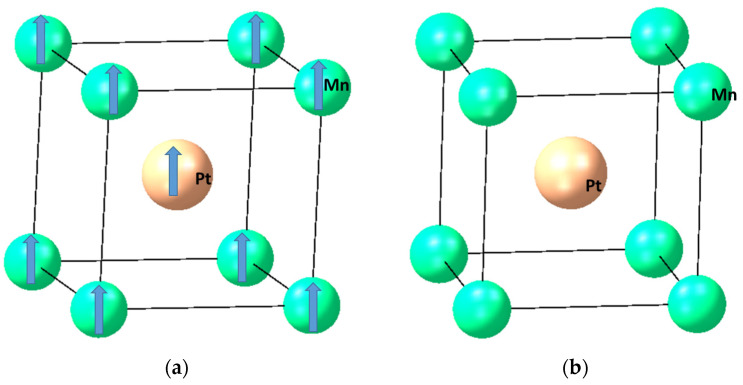
Schematic picture of B2 MnPt in (**a**) ferromagnetic (FM) and (**b**) paramagnetic (PM) MnPt alloy.

The B2 MnPt alloy exists in two magnetic states at high temperatures, namely the paramagnetic (PM) and ferromagnetic (FM) phases. Both crystal structures consist of the same space group and Wyckoff atomic positions. Their main difference is the distance between atoms, i.e., from Mn to Pt and from Mn to Mn as well as from Pt to Pt. For example, FM MnPt has a distance of 2.70 Å from Mn1-Pt1, 3.12 Å from Mn1-Mn1 and 3.12 Å from Pt1-Pt1. In the case of PM, MnPt is 2.60 Å from Mn1-Pt1 and 3.00 Å from Mn1-Mn1, as well as 3.00 Å from Pt1-Pt1. The atomic arrangement of the two systems is the same as shown in Figure 1a,b with spins illustrated for the FM MnPt. The arrows indicate the relative directions of the Mn magnetic moment. The ferromagnetic behavior in Figure 1a is mainly a contribution of Mn atoms, while Pt atoms contribute a small amount of magnetic moment, due to the fact that Pt magnetic moments are significantly smaller than those of Mn. In the case of PM MnPt, both Mn and Pt atoms show no contribution to the magnetic moment, which results in zero.

Furthermore, the obtained lattice parameter of 3.00 Å for PM B2 is in excellent agreement with the theoretical value of 3.01 Å in the literature [25]. Due to the absence of reported data, the predicted lattice parameter of 3.12 Å for FM B2 could not be compared with any results. Since the focus of this work is on the FM B2 MnPt, the results presented below show how the introduction of precious elements (Pd, Ag, Au) affects the lattice parameters, heats of formation, magnetic moments, and elastic properties of the FM B2 MnPt alloy.

**Figure 2:**
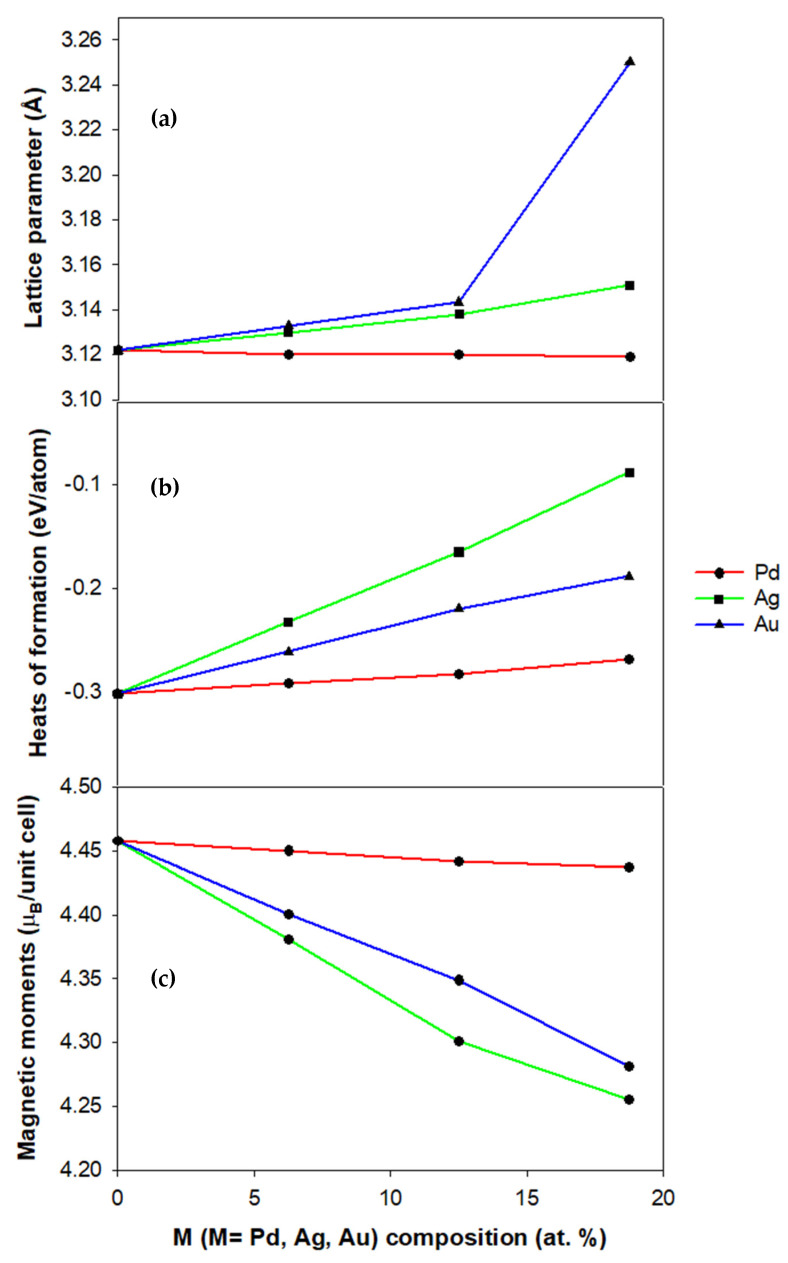
(**a**) Lattice parameters, (**b**) heats of formation and (**c**) magnetic moments against at. % M for B2 FM Mn_50_Pt_50−x_M_x_ alloys (x = 6.25, 12.50 and 18.75).

The computed lattice parameters of B2 Mn_50_Pt_50−x_M_x_ alloys (M = Pd, Ag, Au) (0 ≤ x ≤ 18.75) are displayed in Figure 2a. Notably, the B2 Mn_50_Pt_50−x_M_x_ lattice parameters show a slight decrease with Pd composition but an increase with M content for Ag and Au. This makes sense because Pt’s atomic radius is marginally larger than Pd’s (1.37 Å), but smaller than that of Ag (1.44 Å) and Au (1.44 Å). However, a significant increase in lattice parameter at 18.75 at. % Ag is noted. This behavior could be attributed to the higher coefficient of thermal expansion of Ag. In Figure 2b,c, the calculated heats of formation and the magnetic moments of the Mn_50_Pt_50−x_M_x_ (M = Pd, Au, Ag) alloys along compositions are shown. The thermodynamic stability of B2 Mn_50_Pt_50−x_M_x_ is discussed using the predicted heats of formation (∆*H_f_*) calculated using the following expression:

(1) $${∆H}_{f\;}=E_{C}-\sum_{i}x_{i}E_{i},$$

where the total energy of the system is denoted by $E_{C}$ and the total energy of its constituent elements is represented by $E_{i}$. The lowest negative value (${∆H}_{f}<0$) for the heat of formation is required for a structure to be considered stable, otherwise a positive value (${∆H}_{f\;}>0$) suggests instabilities.

The fact that Figure 2b shows that the B2 Mn_50_Pt_50_ alloy’s heat of formation is negative suggests that there is a good chance that this compound will form.

Despite a slight increase, it is observed that B2 Mn_50_Pt_50−x_Pd_x_ alloy compositions are thermodynamically feasible as indicated by the negative heats of formation for the considered composition range (0 ≤ x ≤ 18.75) as shown in Figure 2b. Furthermore, it is observed that the heat of formation increases with an increase in Ag and Au compositions as shown in Figure 2b. This suggests that the addition of Ag and Au reduces the thermodynamic stability, although the structures can be formed experimentally.

However, a further increase in Pd content results in a slight gradual reduction in magnetic moment, as shown in Figure 2c. The addition of Pd on B2 FM MnPt alloy resulted in the ferromagnetic behavior being maintained. The magnetic moments decrease with an increase in the composition, suggesting that Ag is not good at enhancing the magnetism of MnPt alloy (see Figure 2c). At 18.75 at. % Ag, the structure has lower magnetism for B2 Mn_50_Pt_50−x_Ag_x_. From the magnetism results, it can be distinguished that the ferromagnetic behavior can be observed below 18.75 at. % Ag and above this, antiferromagnetic/paramagnetic behavior can be activated. It is observed that the magnetic moments reduce when Au is introduced to the MnPt alloy. Generally, this work suggests that ferromagnetism is observed but reduces with an increase in Au for 6.25, 12.5 and 18.75 at. %. The precious metal with a smaller atomic radius (Pd) showed an improvement as compared to the elements with a bigger atomic radius i.e., Ag and Au. This suggests that elements such as Pd with their smaller atomic radius can enhance the ferromagnetism of MnPt alloy.

The partial magnetic moments of B2 FM Mn_50_Pt_50−x_M_x_ alloys are highlighted in Table 1. This indicates the magnetic contribution of each atom in the total magnetic moments in the compound. It is observed that Mn atoms contribute more to the total magnetic moments of the binary B2 FM MnPt alloys, as explained above with the Pt atom contributing less, suggesting that indeed the structure shows ferromagnetic behavior. As the amount of Pd is added, it is also observed that Mn atoms contribute more while Pt and Pd contribute almost the same but less due to their atomic size. More interestingly, the contribution of Mn atoms increases slightly, while Pt and Pd atoms reduce with an increase in composition as shown in Table 1.

This suggests that the addition of Pd slightly improves the magnetism of the B2 FM MnPt alloy and that the presence of Mn atoms contributes to the ferromagnetic behavior. This indicates that the directions of electron spins are aligned parallel to each other due to the exchange interaction for all the compositions. In the case of the addition of Ag, it is noted that Mn atoms contribute more to the total magnetic moment as compared to Pt and Au. The contribution decreases with an increase in Ag composition. This suggests that Ag cannot be used to improve the ferromagnetism of B2 FM MnPt alloy as it may transform the system to paramagnetic. When Au is added, the Mn atoms contribute more as compared with the Ag addition. The contribution of Mn atoms decreases with an increase in Au compositions. This suggests that the ferromagnetism is compromised when Mn atoms contribute less. The Au tends to contribute less towards the total magnetic moments. It is noted that the precious elements do not contribute antiferromagnetism on the Pt-site but only ferromagnetism below 18.75 at. % M. The overall observation is that the Pt contribution to the magnetic moment is due to unpaired electrons in the d-orbital, so that when d^10^ is added it reduces the magnetic moment.

### 3.2. Elastic Properties

The mechanical properties of a solid can be obtained through its elastic constants. These characteristics can be used to characterize a crystal’s ability to withstand external stress. Additionally, they offer crucial details regarding bonding properties close to the equilibrium state. Investigating the elastic constants is therefore crucial to comprehending the mechanical behavior of Mn_50_Pt_50−x_M_x_ alloys. Based on energy variation, theoretical elastic constants were computed for the equilibrium lattice configuration under mild strains. A relaxed unit cell lattice structure is subjected to a suitable set of strains. The resulting change in total energy on the deformation is then used to calculate the elastic constants. They can differ depending on the type of lattice i.e., for the cubic lattice there are three (C_11_, C_12_, C_44_) independent elastic constants [26,27]. The elastic properties revealed diverse elastic behavior of the B2 Mn_50_Pt_50_ structure. According to [27], the cubic system’s mechanical stability condition is as follows:

(2) $$C_{44}\;>\;0;\;C_{11}\;>\;C_{12}\;\mathrm{and}\;C_{11}+2C_{12}\;>0,$$

For the structure to be stable, the stability criterion for the elastic constants needs to be met. The following tables and figures present the calculated single-crystal elastic constants for these compounds as reported in this paper.

For practical purposes, mechanical properties like bulk modulus (B), shear modulus (G), Young’s modulus (E), hardness (Hv), and Poisson’s (*v*) ratio can also be calculated using elastic constants. In this paper, Young’s modulus (E) is calculated to estimate the physical stiffness of the Mn_50_Pt_50−x_M_x_ alloys and is presented in the tables below. It is more rigid and less likely to deform if the value of E is higher.

Using the mechanical properties of these compounds, we analyzed their ductility using Poisson’s ratio. Metals with a Poisson’s ratio ≥0.26 are regarded as ductile, whereas metals having a Poisson’s ratio less than 0.26 are referred to as being brittle [28]. One crucial property of materials that can be used to gauge its capacity to withstand localized deformation is its hardness (Hv) [29]. To determine the hardness (Hv), the following calculation can be used:

(3) $$H_{V}=\frac{E(1-2v)}{6(1+v)},$$

where *v* stands for Poisson’s ratio and *E* stands for Young’s modulus. The following tables display the calculated Hv of Mn_50_Pt_50−x_M_x_.

Table 2 presents the calculated elastic constants of B2 Mn_50_Pt_50_ phases along with the available theoretical information. In order to describe the mechanical stability of the binary Mn_50_Pt_50_ alloy, we follow the stability criteria in Equation (2). PM-B2 Mn_50_Pt_50_ appears to have the highest value of C_12_ which is greater than C_11_, and thus leading to C’ being negative, an indication of mechanical instability at 0 K. This is in agreement with experimental data suggesting that B2 PM Mn_50_Pt_50_ is a high-temperature phase. The predicted elastic constants for PM-B2 Mn_50_Pt_50_ confirm the findings from the theoretical view within 5% [24] as shown in the parenthesis. It is noted that FM-B2 Mn_50_Pt_50_ satisfies all the cubic mechanical stability criteria as stipulated in Equation (2).

#### 3.2.1. Mn_50_Pt_50−x_Pd_x_

**Figure 3:**
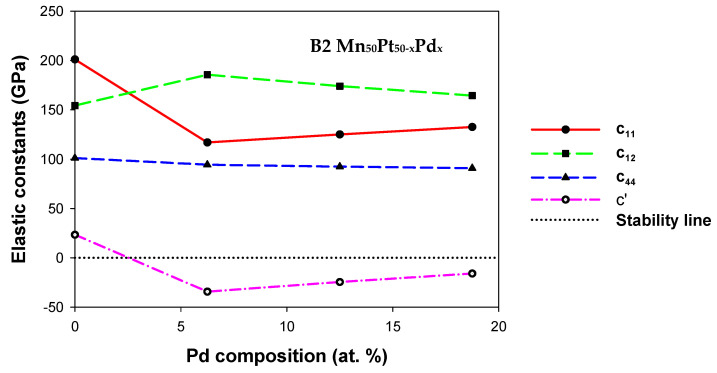
Elastic constants (GPa) of B2 Mn_50_Pt_50−x_Pd_x_ (0 ≤ *x* ≤ 18.75) ternary alloys.

Figure 3 displays the computed elastic constants for the B2 Mn_50_Pt_50−x_Pd_x_ alloys (0 ≤ *x* ≤ 18.75). For the structure to be stable, the stability criterion for the elastic constants needs to be met. Figure 3 illustrates that none of the predicted Cijs for B2 Mn_50_Pt_50−x_Pd_x_ satisfy the stability criteria due to C_11_ being less than C_12_, which produced a negative elastic shear modulus (C′ < 0). A negative C′ on Pd addition signals a possible martensitic transformation (MT).

In order to determine the stiffness of the B2 Mn_50_Pt_50−x_Pd_x_, we calculated Young’s modulus, as shown in Table 3. It is observed that the B2 Mn_50_Pt_50−x_Pd_x_ alloy has lower Young’s modulus value, indicating the least stiffness and the ability to deform conveniently. The findings imply that the addition of Pd does not improve the Mn_50_Pt_50_ alloy’s stiffest point.

Poisson’s ratio (*v*) was used to analyze the ductility of B2 Mn_50_Pt_50−x_Pd_x_ alloys as indicated above. The *v* was greater than 0.26 for B2 Mn_50_Pt_50−x_Pd_x_ alloys, which implies that the structures have ductile behavior. The results suggest that B2 Mn_50_Pt_43.75_Pd_6.25_ is more ductile with the highest values of *v*. For B2 Mn_50_Pt_50−x_Pd_x_ alloys, it is observed that the Hv decreases as Pd increases (0 ≤ x ≤ 18.75). It appears from the findings that adding Pd will not yield the hardest material.

#### 3.2.2. Mn_50_Pt_50−x_Ag_x_

**Figure 4:**
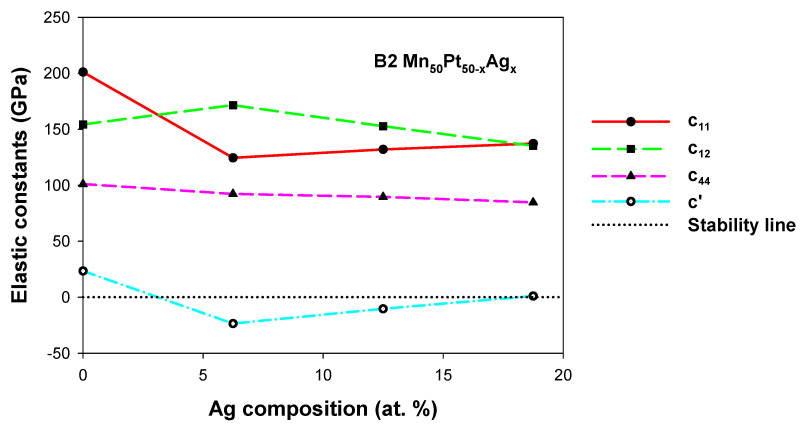
Elastic constants (GPa) of the B2 Mn_50_Pt_50−x_Ag_x_ (0 ≤ x ≤ 18.75) ternary alloys.

The elastic constants of the structures for B2 Mn_50_Pt_50−x_Ag_x_ (0 ≤ x ≤ 18.75) with the addition of Ag are displayed in Figure 4. The elastic constants C_11_, C_12_ and C_44_ are positive for B2 Mn_50_Pt_50−x_Ag_x_, as shown in Figure 4. However, C_11_ decreases with the addition of Ag content while C_12_ increases, suggesting that the structure is becoming mechanically unstable (breaking stability condition, C_11_ > C_12_). This is evident at 6.25 and 12.50 at. % Ag, where C’ is negative, thus rendering the structures mechanically unstable at these concentrations. On the contrary, a slight mechanical stability is observed at 18.75 at. % Ag. The results for 6.25 and 12.50 at. % Ag suggest a possible (MT) due to negative C’ in this composition range.

The predicted Young’s (E) modulus, hardness (Hv) and Poisson’s ratio (v) are shown in Table 4. It can be seen that the E values reduce with an increase in Ag composition for B2 Mn_50_Pt_50−x_Ag_x_ alloys, which means that the structures have less ability to resist elastic deformations.

The ductility and brittleness of the B2 Mn_50_Pt_50−x_Ag_x_ alloys were estimated using Poisson’s ratio (*v*). The results showed that the binary B2 phase does satisfy the stability criteria, as *v* is greater than 0.26. When Ag was introduced, the *v* values were found to be greater than 0.26, which suggests that the structures are ductile.

As shown in Table 4, the values of hardness decrease with an increase in Ag concentration. The addition of Ag was found not to be the best choice to improve the hardness of the Mn_50_Pt_50_ alloy.

#### 3.2.3. Mn_50_Pt_50−x_Au_x_

**Figure 5:**
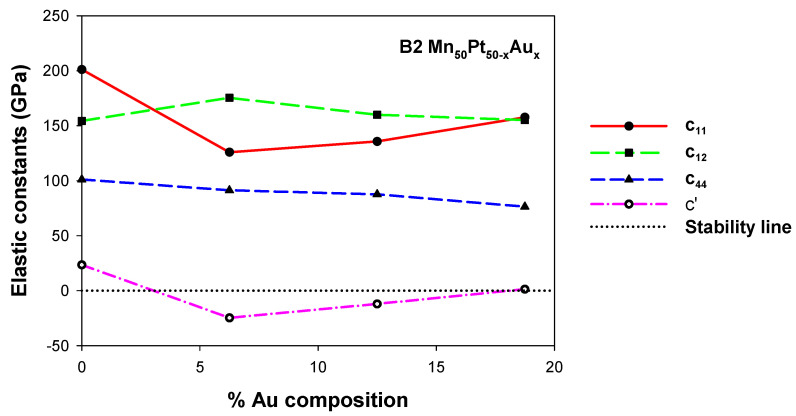
Elastic constants (GPa) of the B2 Mn_50_Pt_50−x_Au_x_ (0 ≤ *x* ≤ 18.75) ternary alloys.

The computed elastic constants for Mn_50_Pt_50−x_Au_x_ alloys (0 ≤ x ≤ 18.75) are displayed in Figure 5. It is observed that for the intended compositions of B2 Mn_50_Pt_50−x_Au_x_ alloys (0 ≤ x ≤ 18.75), all independent elastic constants C_11_, C_12_, and C_44_ are positive. At small Au content (6.25 and 12.50 at. %), the C’ is negative, indicating a potential MT since it makes the structures mechanically unstable at those concentrations. It is noteworthy that above 18.75 at, the elastic shear modulus (C′) exhibits positivity, which indicates that the structures are mechanically stable at high Au content. The results suggest that the Au composition above 18.75 at.% may hinder further transformation to low-temperature phase.

Table 5 shows the calculated Young‘s (E) modulus, hardness (Hv) and Poisson’s (*v*) ratios for B2 Mn_50_Pt_50−x_Au_x_ alloys (0 ≤ x ≤ 18.75). As composition increases, the E values are observed to decrease, indicating that Au has the lowest resistance to elastic deformations. The calculated *v* is greater than 0.26 for B2 Mn_50_Pt_50−x_Au_x_ alloys (0 ≤ x ≤ 18.75) which suggests that the structures are ductile. Furthermore, it is noted that hardness decreases with an increase in Au concentration as shown in Table 5. Due to the small values of Hv, the predicted results showed that Au addition does not have the strongest ability to enhance the hardness of the binary Mn_50_Pt_50_ system.

### 3.3. Vibrational Properties of PM and FM B2 MnPt Structures at 50 at. % Pt

**Figure 6:**
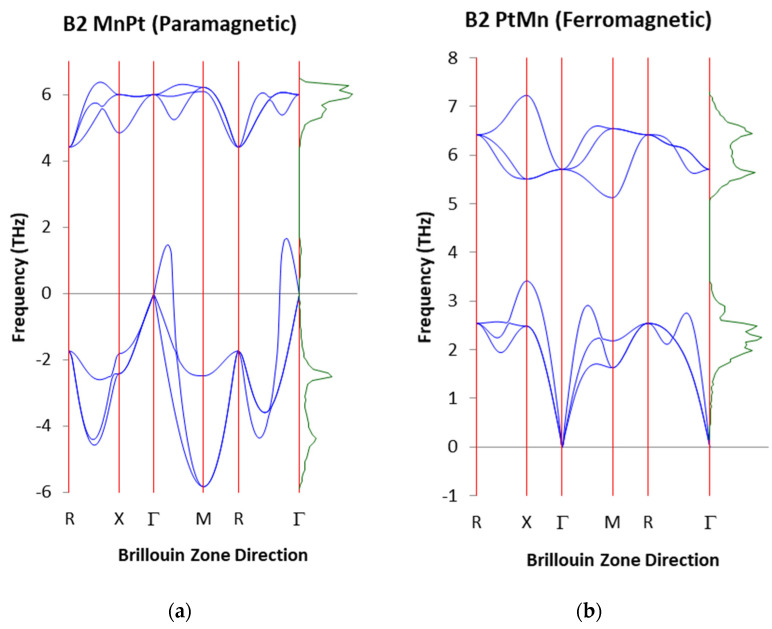
Phonon dispersion curves for (**a**) PM and (**b**) FM B2 MnPt alloy.

Figure 6 displays the computed phonon dispersion curves for the PM and FM MnPt structures. The dispersion curves show two different kinds of phonons: the acoustic and optical modes, which correspond to the lower and upper sets of curves in the diagram, respectively. Since there are soft modes seen, our phonon dispersion calculations confirm that the PM B2 MnPt structure is unstable, as seen in elastic constants (Table 2). The soft modes are observed along the R, X, and M directions. The pure elastic instability C’= ½ (C_11_ − C_12_) < 0 is represented by the downward slope of the acoustic Γ-M branch. These soft modes suggest that the structure is vibrationally unstable with the potential to undergo a phase transition to lower-temperature phases. Furthermore, the FM MnPt structure displays no soft modes (positive frequency) in the phonon dispersion curve which corresponds to the most stable structure behavior as predicted by the elastic constants. This implies that there is no phase transition of the structure to lower temperature phases like L1_0_.

## 4. Conclusions

The lattice parameters, heats of formation, magnetic moments, and mechanical properties of Mn_50_Pt_50−x_M_x_ (M = Pd, Ag, Au) alloys (0 ≤ x ≤ 18.75) were successfully studied for possible spintronic applications using the ab initio DFT approach. The available experimental and theoretical values of lattice parameters showed good agreement with our results, within 5%. Based on calculated heats of formation, current results suggest that thermodynamic stability can be maintained for B2 Mn_50_Pt_50−x_Pd_x_ alloys (0 ≤ x ≤ 18.75) while it is significantly reduced by the addition of Au and Ag. It was revealed that ferromagnetism is maintained with Pd addition but significantly reduced in the case of Au and Ag. The elasticity results of B2 Mn_50_Pt_50−x_Ag_x_ and Mn_50_Pt_50−x_Au_x_ at compositions between 6.25 and 12.50 signal a possible martensitic transformation to a lower symmetry phase due to C’ < 0 but regain mechanical stability at 18.75 at%. On the contrary, the mechanical stability criteria are never satisfied in B2 Mn_50_Pt_50−x_Pd_x_. It was further revealed using the values of the Poisson’s ratio that an increase in Pd, Ag, and Au could effectively improve the ductility of the B2 Mn_50_Pt_50−x_M_x_ compounds.

Pd, Ag, and Au could effectively improve the ductility of the compound.

## Figures and Tables

**Table 1:** The atomic partial magnetic moments (μ_B_/unit cell) of B2 FM Mn_50_Pt_50−x_M_x_ alloys (x = 6.25, 12.50, 18.75 and 25), where M = Pd, Ag, and Au.

Composition	Mn	Pt	M
Mn_50_Pt_50_	3.784	0.667	
	Mn_50_Pt_50−x_Pd_x_		
Mn_50_Pt_43.75_Pd_6.25_	3.788	0.407	0.360
Mn_50_Pt_37.5_Pd_12.5_	3.792	0.398	0.365
Mn_50_Pt_31.25_Pd_18.75_	3.797	0.404	0.356
	Mn_50_Pt_50−x_Ag_x_		
Mn_50_Pt_43.75_Ag_6.25_	3.773	0.411	0.062
Mn_50_Pt_37.5_Ag_12.5_	3.756	0.374	0.060
Mn_50_Pt_31.25_Ag_18.75_	3.758	0.355	0.056
	Mn_50_Pt_50−x_Au_x_		
Mn_50_Pt_43.75_Au_6.25_	3.783	0.401	0.114
Mn_50_Pt_37.5_Au_12.5_	3.780	0.376	0.112
Mn_50_Pt_31.25_Au_18.75_	3.765	0.362	0.096

**Table 2:** Elastic constants of PM and FM MnPt structures at 50 at. % Pt. The theoretical values are given in parentheses.

Elastic Constants, C_ij_ (GPa)	PM-B2 Mn_50_Pt_50_	PM-B2 Mn_50_Pt_50_
C_11_	68 (68)	201
C_12_	330 (329)	154
C_44_	138 (134)	101
C′	−131 (−131)	23

**Table 3:** The calculated Young’s modulus E (GPa), Poisson’s ratio (*v*), and hardness (Hv) (GPa) of Mn_50_Pt_50−x_Pd_x_ alloy.

Structure	Young’s Modulus (GPa)	Poisson’s Ratio	Hardness (GPa)
Mn_50_Pt_50_	184.69	0.32	6.80
Mn_50_Pt_43.75_Pd_6.25_	118.38	0.38	2.90
Mn_50_Pt_37.5_Pd_12.5_	124.90	0.37	3.36
Mn_50_Pt_31.25_Pd_18.75_	128.51	0.36	3.66

**Table 4:** The calculated Young’s modulus E (GPa), Poisson’s ratio (*v*), and hardness (H) (GPa) of Mn_50_Pt_50−x_Ag_x_ alloy.

Structure	Young’s (Gpa)	Poisson Ratio	Hardness (Gpa)
Mn_50_Pt_50_	184.69	0.32	6.80
Mn_50_Pt_43.75_Ag_6.25_	125.43	0.37	3.44
Mn_50_Pt_37.5_Ag_12.5_	133.67	0.35	4.28
Mn_50_Pt_31.25_Ag_18.75_	133.24	0.34	4.66

**Table 5:** The calculated Young’s modulus E (GPa), Poisson’s ratio (σ), and hardness (Hv) (GPa) of Mn_50_Pt_50−x_Au_x_ alloy.

Structure	Young’s Modulus (GPa)	Poisson’s Ratio	Hardness (GPa)
Mn_50_Pt_50_	184.69	0.32	6.80
Mn_50_Pt_43.75_Au_6.25_	123.00	0.37	3.23
Mn_50_Pt_37.5_Au_12.5_	129.53	0.36	3.81
Mn_50_Pt_31.25_Au_18.75_	150.42	0.33	5.53

## Data Availability

Data are contained within the article.
